# The hypothalamic neuropeptide FF network is impaired in hypertensive patients

**DOI:** 10.1002/brb3.229

**Published:** 2014-04-10

**Authors:** Valeri D Goncharuk, Ruud M Buijs, Jack H Jhamandas, Dick F Swaab

**Affiliations:** 1 Netherlands Institute for NeuroscienceAmsterdam ZO, 1105 BA, The Netherlands; 2 Russian Cardiology Research CenterMoscow, 121552, Russia; 3 Division of Neurology, Department of Medicine, University of AlbertaEdmonton, AB, T6G 2S2, Canada; 4 Instituto de Investigaciones Biomedicas, Universidad Nacional Autonoma de MexicoMexico City, 04510, Mexico

**Keywords:** Essential hypertension, hypothalamic cardiovascular regulation, quantitative immunohistochemistry

## Abstract

**Background:**

The human hypothalamus contains the neuropeptide FF (NPFF) neurochemical network. Animal experiments demonstrated that NPFF is implicated in the central cardiovascular regulation. We therefore studied expression of this peptide in the hypothalamus of individuals who suffered from essential hypertension (*n* = 8) and died suddenly due to acute myocardial infarction (AMI), and compared to that of healthy individuals (controls) (*n* = 6) who died abruptly due to mechanical trauma of the chest.

**Methods:**

The frozen right part of the hypothalamus was cut coronally into serial sections of 20 *μ*m thickness, and each tenth section was stained immunohistochemically using antibody against NPFF. The central section through each hypothalamic nucleus was characterized by the highest intensity of NPFF immunostaining and thus was chosen for quantitative densitometry.

**Results:**

In hypertensive patients, the area occupied by NPFF immunostained neuronal elements in the central sections through the suprachiasmatic nucleus (SCh), paraventricular hypothalamic nucleus (Pa), bed nucleus of the stria terminalis (BST), perinuclear zone (PNZ) of the supraoptic nucleus (SON), dorso- (DMH), ventromedial (VMH) nuclei, and perifornical nucleus (PeF) was dramatically decreased compared to controls, ranging about six times less in the VMH to 15 times less in the central part of the BST (BSTC). The NPFF innervation of both nonstained neuronal profiles and microvasculature was extremely poor in hypertensive patients compared to control.

**Conclusions:**

The decreased NPFF expression in the hypothalamus of hypertensive patients might be a cause of impairment of its interaction with other neurochemical systems, and thereby might be involved in the pathogenesis of the disease.

## Introduction

Neuropeptide FF (NPFF), a member of evolutionary highly conserved family of amidated neuropeptides (Yang et al. 1985; Perry et al. 1997; Nichols 2003; López-Vera et al. 2008), is involved in central regulation of a variety of physiological functions including food intake (Sunter et al. 2001; Nicklous and Simansky 2003), fluid balance (Arima et al. 1996; Sunter et al. 2001), and nociception (Yang et al. 2008). Moreover, this neuropeptide has been shown to be a major participant in central cardiovascular regulation. Indeed, intravenous injections of NPFF (Allard et al. 1995; Prokai et al. 2006; Kapel'ko et al. 2009) or microinjections of the peptide either into the lateral cerebral ventricle (Jhamandas et al. 2006; Simonin et al. 2006) or directly into the solitary nucleus (Laguzzi et al. 1996) caused changes in blood pressure and heart rate in the anesthetized and conscious rats. These hemodynamic changes were blocked when NPFF was applied in combination with selective NPFF receptor antagonists (Prokai et al. 2006; Simonin et al. 2006). The chemical anatomy of the NPFF system is well characterized across a range of species including within the human brain. Human genes encoding for both, the NPFF precursor (Perry et al. 1997; Vilim et al. 1999) and two NPFF receptors (Bonini et al. 2000; Elshourbagy et al. 2000; Hinuma et al. 2000; Liu et al. 2001), were cloned and tissue distribution analysis (Bonini et al. 2000) as well as immunocytochemical mapping (Goncharuk et al. 2004, 2006; Goncharuk and Jhamandas 2008) have been performed. NPFF-synthesizing neurons and cells that express NPFF receptors were localized within the human hypothalamus and brainstem. Recently, we have demonstrated a significant decrease in the NPFF innervation of the vagal cardiovascular regulatory centers in the brainstem of hypertensive patients (Goncharuk et al. 2011). As the hypothalamus plays a key role in regulation of brainstem autonomic outflow, in the present study, we carried out a comparative quantitative immunocytochemical analysis of the NPFF network between hypothalami from hypertensive patients, who died due to acute myocardial infarction (AMI) or brain hemorrhage, and those from healthy individuals who died due to mechanical trauma. The data obtained imply a malfunction of the hypothalamic NPFF system in essential hypertension.

## Patients and Methods

### Patients

The hypothalamus of hypertensive patients (*n* = 8) who died suddenly either due to AMI or brain hemorrhage (BH) or from healthy individuals (*n* = 6) who died abruptly due to mechanical trauma (MT), was dissected by routine autopsy 3.5–9 h after the death (Table 1). Systolic (SBP)/diastolic (DBP) blood pressure measured repeatedly in hypertensive patients at least through last 3 years before death was on the average 169.8 ± 2.5/106.6 ± 2.3 mmHg, whereas in healthy individuals of the control group it was 128.0 ± 2.5/85.2 ± 1.3 mmHg (Table 1). Brain samples were collected in Russia in the period 1996–1998 at the peak of social instability and deterioration of medical assistance. Therefore, most hypertensive subjects (*n* = 6), despite the well-documented medical history of their disease, did not receive modern antihypertensive therapy and the only two of the eight (Table 1) were treated systematically with individualized combination of antihypertensives including diuretics, sympathoplegic agents, vasodilators, angiotensin converting enzyme (ACE) inhibitors, and angiotensin receptor antagonists. The control brain samples were obtained from the Institute of Forensic Medicine (Moscow, Russia). The only samples from healthy individuals who died by accident due to mechanical trauma of the chest were collected. The high levels of alcohol, drugs in the blood, or any other similar aggravations were considered as incompatible with our study. Individuals from the control and hypertensive groups were matched by gender, age, time of the death, and postmortem delay (Table 1) to avoid effects of these potential confounding factors on peptide content in our study. Neither hypothalamic lacunar infarctions nor pathological changes causing secondary hypertension were identified either by routine autopsy or following microscopical tissue analysis. Also, no evidence of neurological disease was detected in the brain of individuals studied. Moreover, subjects did not suffer from any mental illness and never took antidepressants. A written informed consent was obtained prior to all autopsies and the subsequent procurement of brain tissue for this research project. This study was approved by the Ethics Committee of the Russian Cardiology Research Center and of the Institute of Forensic Medicine (Moscow, Russia).

**Table 1:** Clinicopathological data

Protocol no	Status	Gender	Age (year)	P.m. delay (hours)	SBP (mmHg)	DBP (mmHg)	Cause of death
1615	con	M	23	3.50	125 (135, 110)	82 (90, 70)	MT
01-2915	con	M	30	4.00	120 (130, 110)	85 (90, 75)	MT
2154	con	M	40	5.00	130 (135, 130)	85 (90, 75)	MT
0109	con	M	49	6.00	130 (137, 128)	82 (87, 85)	MT
1777	con	M	53	4.00	125 (133, 118)	87 (90, 85)	MT
51-1840	con	F	64	4.50	138 (140, 130)	90 (95, 80)	MT
M±SEM			43.17 ± 6.204	4.50 ± 0.365	128.0 ± 2.517	85.17 ± 1.249	
5476	hpt	M	36	9.00	160 (175, 140)	95 (110, 90)	AMI
50-159	hpt	M	38	4.00	170 (200, 154)	110 (127, 95)	AMI
11-1168	hpt	F	48	3.00	168 (195, 150)	108 (115, 95)	BH
391	hpt	M	50	2.67	180 (195, 142)	112 (120, 103)	AMI
1204	hpt	M	58	2.50	165 (170, 145)	105 (115, 90)	AMI
1339	hpt	M	58	9.00	180 (230, 160)	108 (130, 90)	AMI
5478	hpt	M	65	3.50	170 (200, 130)	115 (125, 100)	AMI
38	hpt	F	68	4.75	165 (175, 145)	100 (105, 95)	AMI
M±SEM			52.63 ± 4.153	4.80 ± 0.951	169.8 ± 2.512	106.6 ± 2.299	
*P*			0.2123	0.7975	<0.0001	<0.0001	

Con, control; hpt, hypertension; M, male; F, female; SBP and DBP, systolic and diastolic blood pressure, respectively, presented as median and maximal and minimal values (in parentheses); AMI, acute myocardial infarction; BH, brain hemorrhage; MT, mechanical trauma.

Means (M) of age and postmortem (P.m.) delay did not differ statistically between control and hypertensive groups (*P* = 0.2123, *P* = 0.7975, respectively, unpaired *t*-test).

Both SBP and DBP is higher in hypertensive patients compared to those in control group of healthy individuals (*P* < 0.0001, unpaired *t-*test).

### Immunohistochemistry

Protocols for both the NPFF immunohistochemistry and tests for antibody specificity have been previously described in detail by us (Goncharuk et al. 2011). In brief, the frozen right part of the hypothalamus was cut into serial coronal sections of 20 *μ*m thickness, and each tenth section was taken for a staining procedure. It should be mentioned that sets of serial sections of both hypertensive and control hypothalamus were stained in parallel, under the same conditions and using the same solutions. The floating sections were pretreated with absolute methanol and 3% H_2_O_2_, followed by incubation with the rabbit polyclonal NPFF antibody (1:4000), incubation with biotinylated goat anti-rabbit IgG (H+L) (Vector; 1:400), incubation with ABC (Vector; 1:800) and at last, by incubation in a mixture containing 0.05% 3.3′-diaminobenzidine tetrachloride, 0.2% nickel ammonium sulfate, and 0.001% H_2_O_2_. The final dark blue granular product in cell bodies and processes identified the location of NPFF. The polyclonal rabbit anti-NPFF antibody was provided by Dr. H.-Y.T. Yang (NIMH, Washington, DC) to Dr Fred van Leeuwen (Netherlands Institute for Brain Research, Amsterdam). This antibody (Table 2) showed no cross-reactivity with more than 20 known peptides, including amidated ones (Majane and Yang 1987; Boersma et al. 1993; Goncharuk et al. 2006). The only highly sensitive HPLC-RIA analysis of bovine brain extract demonstrated that this antibody also detected a minor peak in the position of NPAF, an endogeneous octadecapeptide sharing the same C-terminal tetrapeptide with NPFF (Majane and Yang 1987). Moreover, in a previous study, we carried out the preabsorption experiments and demonstrated no cross-reactivity of this NPFF antibody with RFamide related peptide (RFRP-3/NPVF) (Goncharuk et al. 2006). Interestingly, Yano et al. (2003), using specific antibodies against each member of the related RFRP family (RPRF-1 and RPRF-3), and in situ hybridization technique reported different distribution and functional roles for these peptides compared to NPFF. Moreover, using dual immunocytochemistry methods, these authors did not report colocalization of NPFF and RFRP-3, despite NPFF and RPRF-3 sharing the same structure at the C-terminus, Pro-Glu-Arg-Phe-NH2. For a clear delineation of hypothalamic nuclei that stained positively for NPFF, a reference set of adjacent rostro-caudal sections were immunostained with an antibody against vasopressin (VP) and counterstained with cresyl violet. The specificity of VP antibody used (Table 2) has been recently described in detail (Goncharuk et al. 2011).

**Table 2:** Primary antibodies used

Antigen	Immunogen	Manufacturer	Dilution used
Arginine vasopressin	Synthetic peptide Cys-Tyr-Phe-Gln-Asn-Cys-Pro-Arg-Gly-NH_2_ (V-9879, Sigma)	Netherlands Institute for Neuroscience, (Amsterdam), rabbit, polyclonal, #Truus, C.P.180985	1:4000
Neuropeptide FF	Synthetic peptide Phe-Leu-Phe-Gln-Pro-Gln-Arg-Phe-NH_2_ (Pennisula Laboratories,Inc (Belmont, CA)	Kindly provided by Dr H.-Y.T.Yang, National Institute for Mental Health (Washington, DC), rabbit, polyclonal, #1 and #2	1:4000

### Quantitation of data

The hypothalamic nuclei were identified microscopically in immunohistochemically stained sections, taking into account anatomical landmarks such as the third cerebral ventricle wall, optic chiasm, anterior commissure, fornix, optic tract, hypothalamic sulcus, and also using VP- and cresyl violet-counterstained adjacent sections as a guide. The nomenclature is in accordance with the atlas of human brain in stereotaxic coordinates (Mai et al. 2008). Here, we should mention that our preliminary analysis showed that NPFF immunoreactive neuronal profiles and fibers were not evenly distributed within each hypothalamic nucleus. Thus, in rostro-caudal series of coronal sections through the nucleus, the density of NPFF immunoreactivity was usually minimal in the initial and last sections, whereas the highest density was observed, as a rule, in the middle sections. In addition, NPFF immunoreactivity within all hypothalamic nuclei was observed mostly in the form of NPFF fibers. Thus, the number of NPFF neuronal cell bodies was nonnumerous even in the controls, and was extremely low in hypertensive brains. Therefore, for quantitative analysis, we chose one coronal section through the middle of each nucleus, where the highest density of NPFF immunoreactivity was observed. Secondly, in this section, we quantified the entire area covered by both the NPFF immunostained neuronal profiles and the NPFF fibers. The procedure used has been described previously in detail (Goncharuk et al. 2011). In brief, we used an Axioskop microscope with scanning table (Zeiss, Jena, Germany) equipped with a black-and-white CCD camera (Sony XC-77, Tokyo, Japan) that was run by the computer program Image-Pro Plus 6.3 (Media Cybernetics, Bethesda, MD). We recorded a tale digital image of the chosen hypothalamic section under a 40× NeoFluar objective (Zeiss). With this image displayed on a computer screen, we manually outlined the area covered by NPFF neuronal elements. Further, within the defined area, the Image-Pro computer program distinguished depositions of the final product of the immunohistochemical reaction whose optical density was greater than 5× compared to the background. This coefficient was selected by preliminary examination of our preparations, and with its use, the mask counted by the computer program covered the immunostained neuronal elements on the computer screen most precisely. The area of the mask was expressed in mm^2^ and was used for further analysis. The data obtained from the control and hypertensive group were compared statistically using unpaired *t*-test.

### Image processing

Brightfield images were obtained on an Axioplan 2 microscope (Zeiss) with a AxioCamMRc camera (Zeiss), using the MRGrab software package (Zeiss). The digital images were subsequently edited in Adobe Photoshop 7.0 (San Jose, CA) and in the process only brightness and contrast were adjusted.

## Results

**Figure 1:**
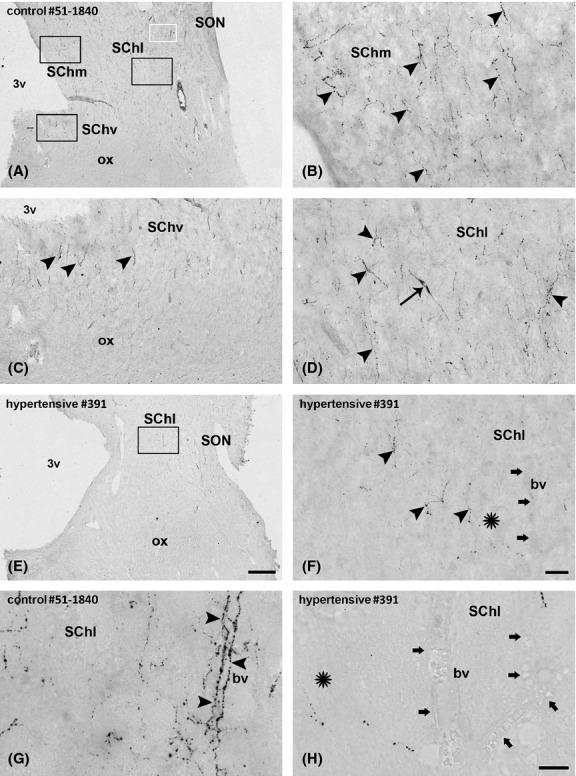
Neuropeptide FF (NPFF) immunostained neuronal profiles and fibers in coronal sections of the suprachiasmatic nucleus (SCh) of the hypothalamus of a control (#51-1840, A–D,G) and a hypertensive patient (#391, E,F,H). In A: boxed areas in the medial (SChm), ventral (SChv), and lateral (SChl) part of the SCh from a control patient are presented at higher magnification in B, C, and D, respectively. The area in the small white box between the SCh and supraoptic nucleus (SON) is shown at higher magnification in G. In E: boxed area within the SChl is shown at higher magnification in F. The blood vessel (bv) within the SChl of a hypertensive patient in F (thick arrows) is presented at higher magnification in H (asterisk in F and H marks the same site in the section). In the SCh of a control patient, a single bipolar NPFF neuron with diameter about 5–7 *μ*m and of typical for SCh neurons morphology (D, arrow) is visible together with numerous NPFF fibers that appear as punctate immunoreactive varicosities and which are mostly oriented along walls of microvessels (B–D, arrowheads). Note the low density of NPFF fibers in the SCh of the hypertensive patient (F, arrowheads). At higher magnification, an extremely high density of double-strained NPFF punctate varicosities is visible along the wall of the microvessel from the SChl in a control patient (G, arrowheads) while such staining is absent in a microvessel of the SChl of a hypertensive patient (H, thick arrows). Calibration bar—0.5 mm in A,E, 10 *μ*m in B–D,F, 20 *μ*m in G,H.

In the most rostral sections of the hypothalamus from controls, we observed individual small NPFF-positive neuronal profiles with a longitudinal diameter of about 5–7 *μ*m and somewhat more numerous NPFF fibers within the suprachiasmatic nucleus (SCh) (Fig. 1A–D). While, at first glance, NPFF neuronal elements were deemed to be equally distributed within various parts of the SCh, further analysis revealed the density of the NPFF fibers in the medial (SChm) and lateral (SChl) parts of the SCh to be elevated compared to the central (SChc) part and even significantly more so when compared to that in the ventral (SChv) part of the nucleus that is partly embedded in the optic chiasm (ox). The NPFF fibers were mostly seen to be associated with the walls of longitudinally running microvessels presumed to represent capillaries based on their caliber (Fig. 1B–D). Sometimes, NPFF immunoreactive varicosities were observed to follow both sides of capillaries for a considerable distance (Fig. 1G). In hypertensive patients, we observed the number of NPFF neurons and fibers within the SCh to be very low (Fig. 1F). Microvessels, too, were poorly innervated by NPFF fibers. Very low levels of NPFF innervation were observed alongside long stretches of such vessels (Fig. 1H). Quantitative analysis revealed that within the SCh of healthy individuals, the largest area covered by NPFF-positive neuronal elements in the middle frontal section was on average 0.030 ± 0.009 mm^2^, whereas in hypertensive patients, these immunopositive profiles covered only 0.009 ± 0.002 mm^2^ in the corresponding area of the SCh (Table 3). Thus, NPFF immunoreactivity in the hypothalamic SCh of hypertensive individuals is at least threefold less than that from controls (*P* = 0.0018).

**Table 3:** Areas (mm^2^) covered by NPFF immunostained neuronal profiles and fibers in hypothalamic nuclei of control (con) and hypertensive (hpt) patients

Protocol no	Status	SCh	PNZ	PaPC+PaD	BSTC	PaPo	DMH	VMH	PeF
1615	con	0.071	0.051	0.036	0.150	0.016	0.132	0.126	0.072
2154	con	0.034	0.051	0.070	0.489	0.194	0.160	0.060	0.061
01-2915	con	0.015	0.048	0.049	0.808	0.158	0.072	0.039	0.033
0109	con	0.024	0.067	0.038	0.251	0.116	0.040	0.072	0.036
51-1840	con	0.014	0.064	0.076	0.490	0.086	0.033	0.018	0.047
1777	con	0.025	0.055	0.169	0.763	0.091	0.068	0.031	0.081
M±SEM		0.030 ± 0.009	0.056 ± 0.003	0.073 ± 0.020	0.492 ± 0.108	0.110 ± 0.025	0.084 ± 0.021	0.058 ± 0.016	0.055 ± 0.008
50-159	hpt	0.008	0.001	0.008	0.010	0.003	0.007	0.005	0.008
11-1168	hpt	0.004	0.009	0.006	0.057	0.023	0.014	0.009	0.004
1204	hpt	0.004	0.004	0.009	0.045	0.016	0.005	0.004	0.010
5476	hpt	0.008	0.006	0.011	0.024	0.010	0.021	0.016	0.002
38	hpt	0.013	0.002	0.006	0.082	0.028	0.022	0.030	0.005
391	hpt	0.006	0.015	0.004	0.019	0.010	0.003	0.002	0.009
1339	hpt	0.008	0.002	0.004	0.014	0.002	0.022	0.011	0.005
5478	hpt	0.019	0.001	0.003	0.017	0.004	0.012	0.004	0.005
M±SEM		0.009 ± 0.002	0.005 ± 0.003	0.007 ± 0.001	0.034 ± 0.009	0.012 ± 0.003	0.013 ± 0.003	0.010 ± 0.003	0.006 ± 0.001
*P*		0.0018	<0.0001	0.0024	<0.0001	0.0007	0.0020	0.0054	<0.0001

BSTC, bed nucleus of the stria terminalis, central part; DMN, dorsomedial nucleus; PaPC, hypothalamic paraventricular nucleus, parvocellular part; PaPo, hypothalamic paraventricular nucleus, posterior part; PNZ, perinuclear zone of the supraoptic nucleus; SCN, suprachiasmatic nucleus; VMN, ventromedial nucleus; M, arythmetical mean; SEM, standard error of arithmetical mean.

The area of NPFF immunoreactive neuronal profiles and fibers in all nuclei studied is much higher in control than in hypertensive group. The data were analyzed statistically using unpaired *t-*test.

**Figure 2:**
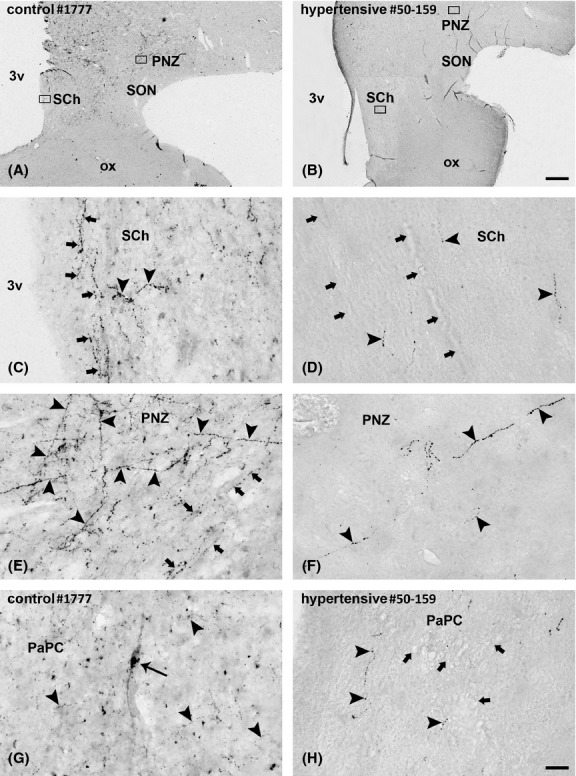
Neuropeptide FF immunostained neuronal profiles and fibers in frontal sections of the hypothalamus of a control (#1777) and a hypertensive patient (#50-159) at the middle level of the supraoptic nucleus (SON). (A) control, boxed area in the suprachiasmatic nucleus (SCh) and boxed area within the perinuclear zone (PNZ) of the SON are shown at higher magnification in C and E, respectively. (B) boxed area in the SCh and PNZ of the hypertensive patient are presented at higher magnification in D and F, respectively. Note in the control patient numerous single strands of NPFF immunoreactive punctate varicosities in the SCh, PNZ and at this level in the hypothalamic paraventricular nucleus, parvocellular part (PaPC) (C, E, and G, respectively, arrowheads). Also notice the strainds of NPFF varicosities innervating both sides of capillaries in the SCh and PNZ (C and E, respectively, thick arrows) and note NPFF neuronal profile in the PaPC (G, arrow). The density of NPFF fibers both in the SCh, PNZ, and PaPC (D, F and H, respectively, arrowheads) is much lower in the hypertensive patient compared to control individual. Note also absence of NPFF innervations of long capillaries within the SCh and PaPC of the hypertensive patient (D and H, respectively, thick arrows). 3v – third ventricle, ox – optic chiasm. Calibration bar—0.5 mm in A,B, 20 *μ*m in C–H.

More caudally, at the middle level of the supraoptic nucleus (SON), we still observed a substantial number of NPFF-positive fibers in the posterior part of the SCh in controls (Fig. 2A,C). No significant NPFF immunostaining was identified in the supraoptic nucleus (SON) proper, whereas the perinuclear zone (PNZ) (immediately adjacent and dorsal to the SON) was characterized by one of the highest concentrations of the NPFF fibers in the control hypothalami (Fig. 2A,E). The capillaries in the SCh at this level (Fig. 2A,C), but also within the PNZ (Fig. 2A,E), were highly innervated by the NPFF fibers. In hypertensive patients, the number of the NPFF-positive neuronal profiles and fibers at this caudal level of the SCh was much less compared to controls (Fig. 2B,D). In addition, in hypothalami of hypertensives, a paucity of NPFF immunostaining was observed within the PNZ (Fig. 2B,F). As well, in hypertensives, an overwhelming majority of capillaries within these structures did not show evidence of close apposition of NPFF fibers as we had observed in controls (Fig. 2 D,F). Quantitative analysis revealed that the largest area of the NPFF immunostaining in PNZ of control individuals was 0.056 ± 0.003 mm^2^ against 0.005 ± 0.003 mm^2^ in hypertensive patients, that is, 11-fold less (*P* < 0.0001) than in controls (Table 3).

Rather sparse and scattered bipolar NPFF neuronal profiles with a diameter of approximately 15 *μ*m (parvocellular neurons) and NPFF fibers of moderate density were observed in the parvocellular (PaPC) (Fig. 2G) and dorsal (PaD) part of the paraventricular hypothalamic nucleus (Pa) of controls. Also here, NPFF-positive fibers as a rule were found to run along a capillary wall or cover nonstained neuronal profiles (Fig. 2G). Nevertheless, an even marked diminution in density of NPFF-positive neuronal elements was detected both in the PaPC (Fig. 2H) and PaD of hypertensive patients. Quantitative analysis showed that on average, this area (PaPC+PaD) was 0.073 ± 0.020 mm^2^ in control individuals and 0.007 ± 0.001 mm^2^, that is, a 10-fold less (*P* = 0.0024), in hypertensive patients (Table 3).

**Figure 3:**
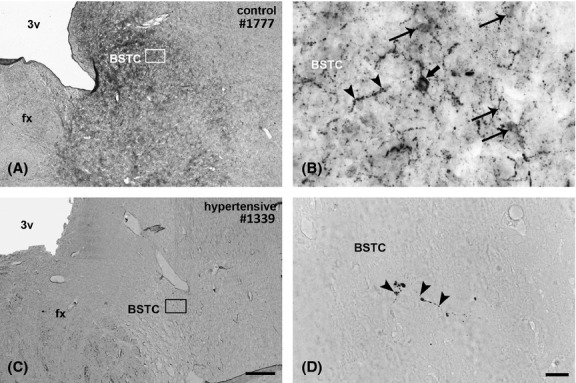
Neuropeptide FF (NPFF) immunocytochemically stained neuronal elements in the central part of the bed nucleus of the stria terminalis, (BSTC) in a control (#1777) and a hypertensive patient (#1339). Boxed area within the BSTC of control (A) and hypertensive (C) patient is presented at high magnification in B and D, respectively. Note in the control patient (B), the presence of a NPFF neuronal profile (thick arrow), numerous NPFF fibers that appear as chains of varicosities (one of such fibers is marked by arrowheads) and many nonstained neuronal profiles surrounded by NPFF presynaptic-like boutons (arrows). In the hypertensive patient (D), NPFF staining is hardly present and is presented here by single NPFF immunostained fiber (arrowheads). 3v – third cerebral ventricle, fx – fornix. Calibration bar—0.5 mm in A,C and 25 *μ*m in B,D

In controls, a very high concentration of the NPFF fibers and some NPFF bipolar neuronal profiles with approximate diameters of 15 *μ*m were detected in the central part of the bed nucleus of the stria terminalis (BSTC) (Fig. 3A,B). In contrast, the concentration of the NPFF-positive neuronal fibers in the BSTC of hypertensive patients was extremely low (Fig. 3C,D). Quantitative analysis showed that the area covered by the NPFF immunoreactivity within the BSTC of hypertensive patients was in average about 15-fold less (*P* < 0.0001) than those in the BSTC of control individuals, that is, 0.492 ± 0.108 mm^2^ and 0.034 ± 0.009 mm^2^, respectively (Table 3).

**Figure 4:**
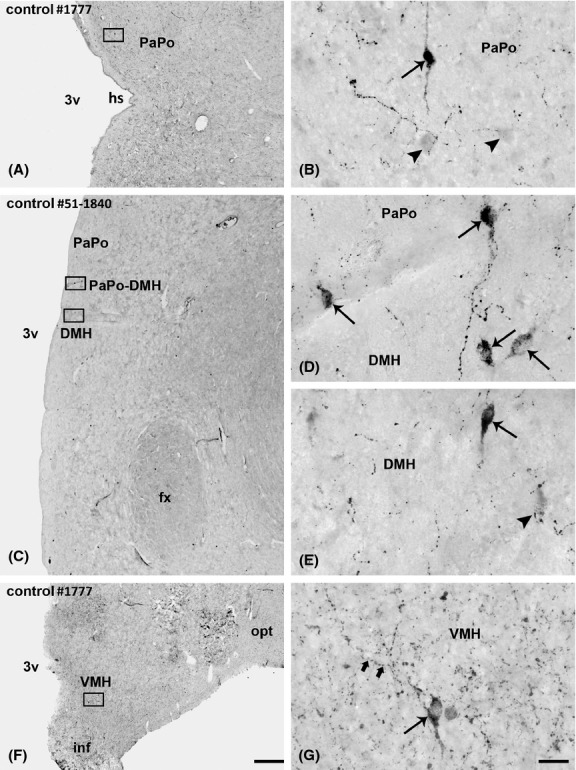
Neuropeptide FF immunocytochemically stained neuronal profiles and fibers in three different fragments of coronal sections from the caudal hypothalamus of two control individuals #1777 (A, F) and #51-1840 (C). Boxed areas in the paraventricular hypothalamic nucleus, posterior part (PaPo) (A), area bordering the PaPo and the dorsomedial hypothalamic nucleus (DMH) (PaPo-DMH) (C), the DMH (C) and the ventromedial part of the ventromedial hypothalamic nucleus (VMH) (F) are shown at higher magnification in B,D,E, and G, respectively. Note the NPFF stained neuronal profiles in the PaPo, PaPo-DMH, DMH, and VMH (arrows in B,D,E,G) and the very high density of NPFF fibers in the VMH (G). Arrowhead in B and E point to non-NPFF stained neuronal profiles covered by NPFF immunoreactive punctate varicosities in the PaPO and DMH, respectively. Thick arrows in G indicate microvessels innervated by NPFF fibers in the VMH. 3v = third cerebral ventricle, fx = fornix, hs – hypothalamic sulcus, inf = infundibular nucleus, opt = optic tract. Calibration bar = 1 mm in A, C, F, and 25 *μ*m in B, D, E, G.

**Figure 5:**
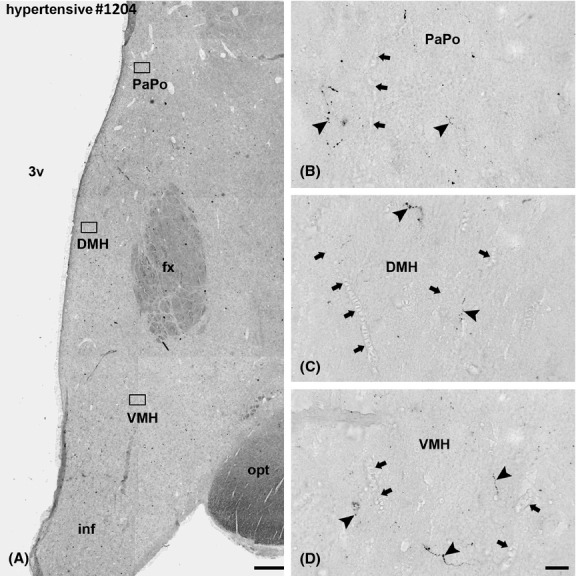
Neuropeptide FF (NPFF) immunostained neuronal fibers in the coronal section of the caudal hypothalamus of hypertensive patient (#1204). Boxed areas within the paraventricular hypothalamic nucleus, posterior part (PaPo), dorsomedial hypothalamic nucleus (DMH), and ventromedial hypothalamic nucleus (VMH) are shown at high magnification in B, C, D, respectively. Note both extremely low density of the NPFF fibers (arrowheads) and a number of non-NPFF innervated capillaries (thick arrows) in all three nuclei – PaPo, DMH, and VMH. 3v – third cerebral ventricle, fx – fornix, inf – infundibular nucleus, opt – optic tract. Calibration bar – 1 mm in A and 25 *μ*m in B–D.

In the caudal hypothalamus of controls, at the level of the infundibular nucleus (inf), both, solitary NPFF bipolar neuronal profiles with a diameter about 15 *μ*m, and much more rarely small groups of two or three such NPFF profiles, were observed in the posterior part (PaPo) of the hypothalamic paraventricular nucleus (Fig. 4B), dorsomedial (DMH) (Fig. 4C,E), and ventromedial (VMH) hypothalamic nucleus (Fig. 4F,G). Interestingly, larger clusters containing 3–4 NPFF-positive neurons were sometimes observed in the most dorsal part of the DMH, bordering the PaPo (Fig. 4C,D). In all these three nuclei, NPFF fibers were on average of moderate density, and were regularly seen to cover nonstained neuronal profiles (Fig. 4B,E) or course along microvessels, presumably capillaries (Fig. 4G). In hypertensive patients, these caudal hypothalamic nuclei—PaPo, DMH, and VMH also demonstrated extremely low density of the NPFF neuronal fibers (Fig. 5B–D). As a result, capillaries innervated by NPFF fibers were hardly ever observed and the noninnervated vessels predominated (Fig. 5B–D). A quantitative analysis revealed that the area occupied by the NPFF immunostained neuronal elements within the PaPo, DMH, and VMH in control individuals was 0.110 ± 0.025 mm^2^, 0.084 ± 0.021 mm^2^, and 0.058 ± 0.016 mm^2^, respectively, whereas in hypertensive patients only 0.012 ± 0.003 mm^2^, 0.013 ± 0.003 mm^2^, and 0.010 ± 0.003 mm^2^ consistent with 9.17 (*P* = 0.0007), 6.46 (*P* = 0.0020), and 5.80 (*P* = 0.0054) -fold less density than in the control group (Table 3).

**Figure 6:**
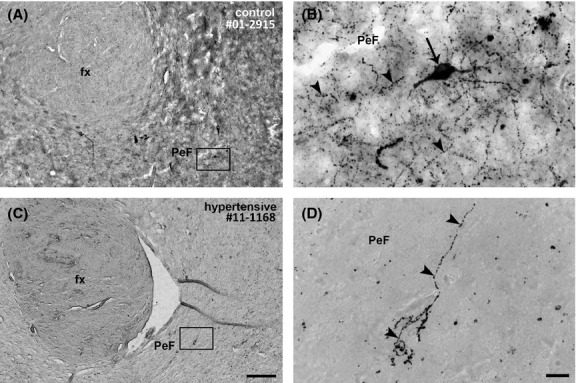
Neuropeptide FF (NPFF) immunocytochemically stained neuronal elements in the perifornical nucleus (PeF) of a control (#01-2915) (A,B) and a hypertensive patient (#11-1168) (C,D). Boxed area in A, C is presented at high magnification in B,D, respectively. Note in the control (B), the NPFF neuronal profile (arrow) and numerous fibers which appear as chains of NPFF positive varicosities (arrowheads). In hypertensive patient (D), only a single NPFF fiber (arrowheads) is present. fx – fornix. Calibration bar – 0.5 mm in A,C, 25 *μ*m in B,D.

Finally, the perifornical nucleus (PeF) in the posterior hypothalamus contained NPFF neuronal profiles and was strongly innervated by NPFF fibers in control individuals (Fig. 6A,B). At the same time, only few NPFF-positive fibers (Fig. 6 C,D) were observed in the PeF of hypertensive patients. The area covered by NPFF immunostained neuronal elements in the control PeF was on average 0.055 ± 0.008 mm^2^,whereas in the PeF of hypertensive patients, this was 0.006 ± 0.001 mm^2^ which is more than ninefold less compared to controls (*P* < 0.0001, Table 3).

## Discussion

In this study, our observations concerning the distribution of hypothalamic NPFF in healthy individuals match those reported for human NPFF receptors (hFF1 and hFF2) (Goncharuk et al. 2004; Goncharuk and Jhamandas 2008) and their respective mRNAs (Bonini et al. 2000; Elshourbagy et al. 2000). A similar localization of NPFF-positive neurons (Aarnisalo and Panula 1995; Vilim et al. 1999; Goncharuk et al. 2006), NPFF-binding sites (Gouarderes et al. 2004), and mRNA for NPFF and its receptors (Vilim et al. 1999; Bonini et al. 2000; Liu et al. 2001; Kalliomäki and Panula 2004) has been reported in the rat hypothalamus. However, in healthy individuals comprising the control group, neuronal NPFF cell bodies and the more numerous NPFF-expressing fibers are diffusely distributed throughout the rostro-caudal axis of the hypothalamus compared to the rat (see also Goncharuk et al. 2006), which reflects well-known differences in segregation of homological hypothalamic nuclei between two species (Koutcherov et al. 2000; Swaab 2003). Moreover, we did not observe any differences in the distribution of NPFF between male and female hypothalami, which is in keeping with our previous observations (Goncharuk et al. 2006).

Interestingly, all of the nuclei that demonstrate detectable NPFF immunoreactivity including the suprachiasmatic nucleus (SCh), perinuclear zone (PNZ) of the supraoptic nucleus (SON), hypothalamic paraventricular nucleus (Pa), bed nucleus of the stria terminalis (BST), dorso- (DMH) and ventromedial (VMH) hypothalamic nucleus and perifornical nucleus (PeF) are known from animal experiments to play an important role in central cardiovascular regulation (Eilam et al. 1991; Cunningham et al. 2002; Marsh et al. 2003; Scheer et al. 2003; Smith and Astley 2007; Horiuchi et al. 2008; Pyner 2009; Nasimi and Hatam 2011). In this study, we observed the NPFF neurochemical network in these nuclei to be dramatically reduced in hypertensive patients.

The interaction of NPFF neuronal network with other neurochemical systems is poorly understood in the human hypothalamus. We have previously shown that many neurons expressing NPFF receptors (FF1, FF2) in the human hypothalamic nuclei morphologically represent local circuit interneurons (Goncharuk et al. 2004; Goncharuk and Jhamandas 2008). A significant portion of these appear to be GABAergic. Indeed, GABAergic interneurons have been shown in the rat to express both types of specific NPFF receptors (FF1, FF2) (Wu et al. 2010) and these receptors can be activated by NPFF to regulate the excitability of magnocellular and parvocelullar hypothalamic Pa neurons (Jhamandas et al. 2007). In addition, it is important to underscore that GABA is the most abundant hypothalamic neurotransmitter and is deemed to play an essential role in regulating a diverse set of autonomic functions that are attributed to the hypothalamic regions including the SCh, PNZ, Pa, BST, DMH, VMH, and PeF (Decavel and Van den Pol 1990; Martin and Haywood 1993; Singewald et al. 1993; Herbison 1994; Zhang and Patel 1998; Liu and Reppert 2000; Wang et al. 2003; Yang and Coote 2003; Li and Pan 2006; Sajdyk et al. 2008; Matsuki et al. 2009; Yi et al. 2009; Han et al. 2010; Yu et al. 2010).

Thus, we can hypothesize that a lack of regulatory effect of NPFF on GABAergic function, for example, in the SCh, may underlie the impairment of neuropeptide expression that we have previously demonstrated in hypertensive patients (Goncharuk et al. 2001) and which might to some extent explain the disturbances in circadian rhythmicity of many physiological parameters characteristic for the hypertensive state (Dyer et al. 1987; Bianchi et al. 1994; Nakano et al. 2001; Grassi et al. 2012). Moreover, reduced NPFF release both in the parvocellular (PaPC), dorsal (PaD) and posterior (PaPo) part of the Pa in hypertensive patients might modulate, in a complex manner, GABAergic inhibitory input to neurosecretory and autonomic Pa neurons, and thereby contribute to increased expression of corticotropin-releasing hormone (CRH) in hypertensive patients that we have previously reported (Goncharuk et al. 2002, 2007). The impairment of NPFF-GABA output within the rest of nuclei—the PNZ-SON, BST, Pa, DMH, VMH and PeF might also be involved in the baroreflex failure (Jhamandas and Renaud 1986; Grassi et al. 2006), increase of blood pressure and heart rate (DiMicco et al. 1996; Takenaka et al. 1996; Zaretskaia et al. 2008; Johnson and Shekhar 2012) in hypertensive patients.

Although we have highlighted here the interactions of NPFF with the GABAergic system, it is likely that the NPFF modulation of central cardiovascular function involves also other neurotransmitter networks. Of these, NPFF interactions with the opioid sytem are perhaps the best studied. Indeed, the NPFF has been previously demonstrated to bind to the delta-opioid receptor (Änkö and Panula 2005), and through this interaction, modulate presynaptically, excitatory synaptic transmission (Chen et al. 2000). In its turn, the delta-opioid receptor is known to be expressed both in the human (Simonin et al., 1994) and animal (Mansour et al. 1987; May et al. 1989; Desjardins et al. 1990; Byku et al. 2000) hypothalamus and was shown in animal experiments to be involved in cardiovascular regulation (Feuerstein and Faden 1982; Kiritsy-Roy et al. 1989; May et al. 1989). Collectively, these observations suggest that hypothalamic NPFF could effectively control centrally generated cardiovascular responses both directly, through specific FF1, FF2 receptors, but also indirectly, modulating delta-opioid-mediated hemodynamic responses.

In addition, the role of NPFF in neurovascular coupling within hypothalamic nuclei deserves comment. Certainly, in control subjects, we observed a dense and robust NPFF innervation of capillaries within the hypothalamus. However, in hypertensive patients, an overwhelming majority of hypothalamic capillaries were devoid of NPFF innervation. In the rat, blood flow control has been shown to be initiated predominantly at the level of the capillaries (Stefanovic et al. 2008) and capillary diameter was found to be regulated by GABAergic interneurons affecting the function of pericytes (Peppiatt et al. 2006). Thus, in hypertensive patients, a lack of NPFF modulation of GABAergic control of microcirculation could compromise blood supply to neurons located within cardiovascular regulatory centers.

In conclusion, we have observed a marked reduction in NPFF in the hypothalamus of hypertensive patients compared to matched controls. We surmise that hypofunction of the NPFF network in key cardiovascular hypothalamic nuclei results in deficient interactions of this peptide with other major transmitter systems, for example, GABAergic one. Moreover, it is entirely possible that NPFF interactions with other neurochemical networks, such as opioid system, or others yet to be elucidated play an important role in brain regulation of cardiovascular and neuroendocrine function. Whether decreased NPFF expression is a cause or consequence of hypertension remains to be addressed, but the present study provides an impetus to examine the role of this peptide in the development of hypertension in animal models of this disease.

## Study Limitations

Despite our efforts to obtain brain samples for the control group that matched those of the hypertensive group as closely as possible, the age distribution was somewhat wider in the control group. Statistical analysis, however, did not reveal a significant difference in the mean age (M) between the control and hypertensive groups. At the same time, the lowest (23 years) and the highest age (68 years) were in the control and hypertensive group, respectively. Unfortunately, the effect of aging on the expression of NPFF was not studied either in the human hypothalamus or in the hypothalamus of experimental animals. However, a similar study was carried out on expression of another hypothalamic neuropeptide—vasopressin (Swaab et al. 1987). This study demonstrated that the number of vasopressin (VP) neurons in the suprachiasmatic nucleus (SCh) from a group of individuals aged 41–60 years—and even more so from a group of 61–80 years old—was markedly higher than those from the group aged 21–40 years. This was true in both males and females. On the other hand, the statistical analysis we carried out in our study (data not presented) did not reveal a correlation between age and the area covered by NPFF immunostained neuronal profiles and fibers in any hypothalamic nucleus either in the control or the hypertensive group. Thus, we assumed that a marked decrease of NPFF expression in the hypothalamus of hypertensive patients would be connected to the disease rather than to the age.

It should be mentioned that we were unable to use Western blot or ELISA techniques to determine the absolute peptide content, because we only had paraformaldehyde-fixed tissue at our disposal. As an alternative, we used quantitative immunohistochemistry to compare relative staining for NPFF in the hypothalamus between hypertensive and control samples. Measurements of optical density in the final DAB product by immunohistochemical determination of the peptide content was shown previously to correlate significantly with the amount of peptide as revealed by radioimmunoassay (Van der Sluijs et al. 1987, 1988). Moreover, we demonstrated before that increased number of CRH-immunostained neurons together with enhanced CRH immunostaining correlated well with increased CRH mRNA radioactive labeling (Goncharuk et al. 2002). Therefore, we feel justified to make the conclusion that the overall content of NPFF in the hypothalamus of hypertensive patients is significantly decreased as compared with control. One important benefit of quantitative immunohistochemistry in the present study was that we could characterize differences in the NPFF content within each nucleus of the hypertensive hypothalamus relative to the same nuclei in the control. Furthermore, microscopic analysis of immunostained sections had the additional advantage of allowing us to observe peculiarities in the interaction of NPFF neuronal cells within specific nuclei as well as dramatic changes in the NPFFergic innervation of the microvasculature in hypertensive patients.
